# Impact of continuous care based on multidisciplinary collaboration on the quality of life of patients with colorectal cancer undergoing chemotherapy

**DOI:** 10.3389/fmed.2026.1799635

**Published:** 2026-04-24

**Authors:** Nan Zhang, Juan Cheng, Shan Wang, Hui Huang

**Affiliations:** Department of Gastrointestinal Surgery, Suqian Hospital of Jiangsu Provincial People's Hospital, Suqian, China

**Keywords:** colorectal cancer, chemotherapy, multidisciplinary collaborative continuous care model, self-management, quality of life

## Abstract

**Aim:**

Colorectal cancer (CRC) patients undergoing chemotherapy face multifaceted challenges post-discharge, including symptom burden, psychological distress, and fragmented care. Conventional follow-up often inadequately addresses these holistic needs. This study aimed to evaluate the efficacy of a structured multidisciplinary collaborative continuous care model compared to routine care in this population.

**Methods:**

A total of 120 CRC patients receiving chemotherapy were randomly assigned to either a study group or a control group. The control group received routine continuous care. The study group received a comprehensive intervention, which included the establishment of five specialized teams (Clinical, Resource, Follow-up, Volunteer, and Rehabilitation Club teams). The intervention encompassed individualized nutrition and symptom management, structured health education (including chemotherapy, ostomy care, and vascular access), proactive follow-up via an electronic system and social media groups, psychological counseling, peer support activities, and home visits. Outcomes were assessed at baseline and 3 months post-intervention using validated scales for gastrointestinal function, cancer-related fatigue, psychological resilience, self-management efficacy, quality of life, incidence of chemotherapy-related adverse reactions, and medication adherence.

**Results:**

After the 3-month intervention, the study group demonstrated significantly greater improvements than the control group across all measured outcomes. Specifically, the study group showed lower GSRS scores (indicating better gastrointestinal function), lower CFS scores (less fatigue), higher CD-RISC scores (greater resilience), higher SUPPH scores (improved self-efficacy), and higher WHOQOL-BREF scores (enhanced quality of life). Furthermore, the study group had a significantly lower incidence of chemotherapy-related adverse reactions and better medication adherence. All improvements were observed over the 3-month study period; longer-term effects remain to be determined.

**Conclusion:**

The implementation of a multidisciplinary collaborative continuous care model significantly improved physical symptoms, psychological well-being, self-management capabilities, treatment tolerance, adherence, and quality of life in CRC patients undergoing chemotherapy over a 3-month follow-up period. These findings demonstrate the short-term effectiveness of this model for post-discharge supportive care. However, longer-term follow-up is needed to assess durability of these benefits and impact on clinical outcomes such as treatment completion, rehospitalization, and recurrence. This model provides an effective, holistic framework for optimizing supportive care during the critical post-discharge period.

**Clinical trial registration:**

Identifier, 2025-SR-0021.

## Introduction

Colorectal cancer (CRC) is a prevalent malignancy of the gastrointestinal tract (1). While radical surgery remains the cornerstone of treatment, adjuvant chemotherapy is often required to eliminate residual disease and improve long-term outcomes (2). Although chemotherapy is essential for prolonging survival, it is frequently associated with significant adverse effects that impair patients’ physical and mental well-being, contributing to cancer-related fatigue and diminished quality of life (3). During hospitalization, patients receive substantial professional support; however, after discharge, their need for comprehensive care remains high. Conventional follow-up approaches often prioritize the management of physical symptoms, overlooking the critical influence of psychological and social factors on overall recovery and quality of life. As a result, they fail to address the holistic needs of post-discharge CRC patients undergoing chemotherapy (4). Evidence has suggested that effective post-treatment care should encompass not only symptom management but also home-based health support, family-centered emotional assistance, and structured extended services (5).

Multidisciplinary collaborative continuous care—a model grounded in the biopsychosocial framework—coordinates interventions through a structured team approach. This strategy has shown promise in enhancing physical and psychological health, reducing cancer-related fatigue, and improving quality of life in cancer populations (6). Nevertheless, current research remains limited regarding its specific effect on the quality of life in CRC patients receiving chemotherapy. Therefore, this study aims to investigate the impact of a multidisciplinary collaborative continuous care model on the quality of life in CRC patients undergoing chemotherapy, thereby providing evidence to optimize integrative supportive care in this population.

## Methods

### Study design

A total of 120 patients with CRC who underwent chemotherapy at our hospital between January 2023 and December 2024 were enrolled in this study.

*Inclusion criteria*: (1) Diagnosis of CRC confirmed by imaging and histopathological examination; (2) Karnofsky Performance Status (KPS) score ≥ 60 (7); (3) Expected survival time > 6 months; (4) Receiving chemotherapy for the first time.

*Exclusion criteria*: (1) Severe dysfunction of major organs (e.g., heart, liver, or kidney); (2) Presence of chemotherapy-related severe complications; (3) Diagnosis of psychiatric disorders or Alzheimer’s disease that could interfere with study participation.

This study was approved by the hospital’s medical ethics committee, and all patients and their families signed the informed consent form.

### Sample size calculation

Prior to study initiation, sample size was calculated using PASS software (version 15.0). The calculation was based on the primary outcome measure (WHOQOL-BREF physical domain score) from a pilot study of 20 CRC patients (10 per group), which showed a mean difference of 8.5 points with a standard deviation of 10.2 between the two groups. To detect a clinically meaningful difference with a two-sided significance level (*α*) of 0.05 and power (1-*β*) of 0.80, a minimum of 48 patients per group was required. Accounting for an anticipated 20% dropout rate, we aimed to enroll 60 patients per group, for a total sample size of 120 patients.

### Data collection at baseline

At enrollment, the following baseline clinical variables were systematically collected from medical records and patient interviews: Demographic and anthropometric data: age, sex, BMI, educational level; Disease-related variables: TNM stage (I–IV), postoperative interval (days from surgery to first chemotherapy), ostomy status (presence or absence of intestinal stoma); Treatment-related variables: chemotherapy regimen (FOLFOX, FOLFIRI, XELOX, or other), number of planned chemotherapy cycles, number of completed cycles at baseline, use of vascular access device (PICC, PORT, or peripheral vein); Comorbidities: hypertension, diabetes, cardiovascular disease, chronic kidney disease, and other chronic conditions, summarized as Charlson Comorbidity Index (CCI) score; Nutritional status: body mass index (BMI, kg/m^2^), serum albumin level (g/L), and weight loss percentage in the past 3 months.

### Randomization, allocation concealment, and blinding

Sequence generation: Patients were randomly allocated to either the study group or the control group in a 1:1 ratio using a computer-generated random number sequence. The random allocation sequence was generated by a biostatistician who was not involved in participant recruitment, intervention delivery, or outcome assessment. A simple randomization method without blocking or stratification was used, as baseline characteristics were expected to be balanced with a sample size of 120.

Allocation concealment: The generated allocation sequence was sealed in sequentially numbered, opaque, sealed envelopes by a research assistant not involved in the study. The envelopes were stored in a locked cabinet accessible only to the designated unblinded staff member. After baseline assessments were completed, the envelopes were opened by a designated unblinded research coordinator to reveal group assignment, ensuring allocation concealment.

Participant enrollment and assignment: Eligible participants were enrolled by a research nurse who screened potential participants against the inclusion/exclusion criteria, obtained informed consent, and conducted baseline assessments. This research nurse was blinded to the allocation sequence until after the baseline assessment was completed. Following baseline assessment, the research nurse contacted the unblinded research coordinator, who opened the next envelope in sequence and informed the research nurse of the group assignment.

Blinding: Due to the nature of the intervention (multidisciplinary collaborative continuous care involving active patient engagement), blinding of participants and nursing staff delivering the intervention was not feasible. However, the following personnel were blinded to group allocation: (1) the research nurse who conducted baseline assessments (until after assessment completion); (2) all outcome assessors (as described in the Outcome Assessment Procedures section); (3) the statistician who performed the final data analysis (the dataset was coded with group A vs. B, with the key revealed only after the analysis was completed).

## Methods

The control group received routine continuous care. During the patient’s hospitalization period, the responsible nurse provided basic nursing services to the patient and carried out various interventions as per the doctor’s instructions. Before the patient was discharged, the nurse instructed the patient on proper diet, medication, rest, maintenance methods and precautions for the intestinal stoma, and informed the patient of the necessity and timing of the follow-up visit. No additional structured follow-up or multidisciplinary input was provided beyond this routine discharge instruction.

The study group received multidisciplinary collaborative continuous care in addition to routine continuous care. The intervention was manualized with predefined protocols for each component. To ensure standardization, all team members received a 4-h training session on the intervention protocol prior to study initiation, followed by a certification test (passing score ≥85%). Monthly quality control meetings were held to review implementation fidelity. The specific measures were as follows:

### Establishment of a professional continuous care team

(1) The Clinical team was established, composed of six gastrointestinal surgery nurses at the N2 level or above. Their responsibilities included assessing patient care needs, developing and implementing nursing measures. Prior to discharge, patient information was transferred to the follow-up team to facilitate subsequent monitoring.
(2) The Resource Team was established, consisting of one intravenous infusion therapist, one cancer pain control specialist, one nutritionist, one oncology specialist nurse, one psychological counselor, and two attending physicians.
(3) The Follow-up team was established, composed of two community nurses.
(4) The Volunteer team was established, consisting of two oncology surgery nurses.
(5) The Rehabilitation club team was established, comprising four rehabilitation club staff members.

### Implementation of continuous care intervention

(1) Clinical team nursing interventions:

*Frequency and duration*: Interventions were delivered daily during hospitalization (average length of stay: 6.3 ± 1.8 days) and once on the day of discharge (a 30–40 min session).

*Actual delivery*: All 60 patients in the study group received the full protocol as planned (completion rate: 100%). Specific interventions included: Dietary guidance: delivered once during hospitalization and reinforced at discharge; Chemotherapy health education: one 20-min structured session; Ostomy care guidance: one 30-min hands-on session with return demonstration; Discharge booklet and WeChat electronic version: provided to all 60 patients (100%).

(2) Follow-up team interventions:

*Frequency*: First telephone follow-up: within 24 h post-discharge (completed for 60/60 patients, 100%); Subsequent telephone follow-ups: at weeks 2, 4, 8, and 12 post-discharge (completion rates: 100, 100, 98.3, and 96.7%, respectively); WeChat/QQ group messaging: weekly health education posts (12 posts total over 3 months).

*Actual engagement*: All 60 patients (100%) joined the WeChat and QQ groups. Average patient-initiated messages per week was 3.2 (range 0–12). The proportion of patients who actively read the weekly posts (defined as clicking “like” or replying) was 78.3% on average across the 12 weeks.

(3) Resource team interventions:

*Frequency*: One theoretical or practical course weekly (12 courses total over 3 months). Each course lasted 45–60 min.

*Actual delivery*: All 12 planned courses were delivered (completion rate: 100%). Course topics included nutrition, chemotherapy side effects, rehabilitation exercises, and catheter care. Video recordings of all 12 courses were pushed to the WeChat/QQ groups, with an average view rate of 71.2% within one week of posting.

For complex nursing problems, additional follow-up guidance was arranged on an as-needed basis. A total of 18 such consultations were provided to 14 patients (23.3%) over the 3-month period.

(4) Volunteer team interventions:

*Frequency*: Home visits were conducted once every two weeks (6 visits per patient over 3 months).

*Actual delivery*: Of the 360 planned home visits (60 patients × 6 visits), 342 were completed (completion rate: 95.0%). Reasons for missed visits included patient refusal (*n* = 12, 3.3%) and scheduling conflicts (*n* = 6, 1.7%). Each home visit lasted approximately 30–45 min and included health education and needs assessment.

(5) Rehabilitation club team interventions:

*Frequency*: Psychological counseling activities (sandplay, yoga, face-to-face interactions): weekly (12 sessions total); Fellowship meetings for post-operative CRC patients: monthly (3 sessions total).

*Actual delivery*: All 12 psychological counseling sessions were held (100% completion). Average attendance per session was 42.3 patients (70.5% of the study group); All 3 fellowship meetings were held (100% completion). Average attendance was 38.7 patients (64.5% of the study group).

*Intervention period*: All components were delivered over a 3-month period (from discharge to 3 months post-discharge).

### Summary of intervention adherence and quality control

To ensure fidelity, the following quality-control procedures were implemented: Each team maintained a structured log documenting the date, duration, content, and patient participation for every intervention encounter. A research assistant not involved in intervention delivery randomly audited 20% of the logs against recorded activities (e.g., telephone records, WeChat posts, home visit sign-in sheets); concordance was 96.3%. Monthly multidisciplinary team meetings were held to review implementation progress, address barriers, and ensure consistency across teams.

Supplementary Table S1 provides a complete summary of intervention components, planned frequency, actual completion rates, and patient engagement metrics.

### Outcomes

The gastrointestinal function was evaluated using the Gastrointestinal Symptom Rating Scale (GSRS) (8). The scale consists of two dimensions: abdominal symptoms (9 items) and defecation status (7 items). It includes 16 symptoms such as abdominal pain, chest discomfort, acid reflux, and hunger, each rated on a 1 to 7 scale. The higher the score, the more severe the symptoms.

The Cancer Fatigue Scale (CFS) was used to evaluate cancer-related fatigue (9). This scale consists of 3 dimensions: cognitive fatigue, emotional fatigue, and physical fatigue, with a total of 15 items. Each item is scored from 1 to 5, and the total score ranges from 15 to 75. The higher the score, the more obvious the cancer-related fatigue of the patient.

The psychological resilience was analyzed through the Connor David-Resilience Scale (CD-RISC) (10), which consisted of 25 items, covering three dimensions: resilience, strength, and optimism. The scores for each dimension ranged from 0 to 52, 0 to 32, and 0 to 16, respectively. The higher the score, the better the psychological resilience of that dimension.

The self-management efficacy was evaluated using the Strategies Used by People to Promote Health (SUPPH) Scale (11). The scale consists of three dimensions: the positive attitude dimension (ranging from 21 to 105 points), the self-decision dimension (ranging from 3 to 15 points), and the self-decompression dimension (ranging from 5 to 25 points). The higher the score, the higher the self-management efficacy.

The World Health Organization Quality of Life Scale-Brief (WHOQOL-BREF) was applied to evaluate the quality of life (12). This scale consists of 4 dimensions: the physical domain, the psychological domain, the environmental domain, and the social relationship domain. Each dimension is scored from 0 to 100, with higher scores indicating a higher level of quality of life.

The incidence of chemotherapy-related adverse reactions in both groups within 3 months was statistically analyzed, including leukopenia, gastrointestinal reactions, and liver dysfunction.

The medication adherence of patients was evaluated using the Morisky Adherence Questionnaire (13). The total score was 4 points. A score of 0 indicated complete adherence, 1 to 3 points indicated basic adherence, and 4 points indicated non-adherence. The higher the score, the lower the medication adherence of the patients. Medication adherence rate = (Number of complete adherents + Number of basic adherents) / Total number × 100%.

### Outcome assessment procedures

To minimize detection bias, a clear separation was maintained between intervention delivery and outcome assessment. Specifically: The Follow-up team was responsible for two distinct types of activities: (a) routine follow-up communication (telephone calls, WeChat/QQ group management) as part of the intervention, and (b) outcome assessment at 3 months. These two functions were performed by different staff members within the Follow-up team to avoid potential bias. The staff member who conducted the 3-month outcome assessment was not involved in any prior follow-up communication with the same patient. All outcome assessors were blinded to group allocation. The assessor received only the patient’s study ID number without any information about whether the patient belonged to the study group or the control group.

All patient-reported outcome scales (GSRS, CFS, CD-RISC, SUPPH, WHOQOL-BREF, and Morisky Medication Adherence Questionnaire) were completed independently by patients in a quiet, private room without the presence of any nursing staff or assessor. Patients placed the completed questionnaires into a sealed opaque envelope, which was then collected by a research assistant who was not involved in either intervention delivery or outcome assessment.

For the incidence of chemotherapy-related adverse reactions (leukopenia, gastrointestinal reactions, liver dysfunction), these data were extracted from electronic medical records by a research assistant blinded to group allocation, based on pre-specified criteria.

A standardized assessment protocol was developed prior to study initiation. All outcome assessors completed a 4-h training session on the standardized administration of each scale, followed by a certification test (passing score ≥90%). Inter-rater reliability was assessed using 10 pilot patients, with an intraclass correlation coefficient (ICC) of 0.94 (95% CI: 0.88–0.97) for the total scores across all scales.

Supplementary Table S2 provides a detailed summary of the outcome assessment procedures, including who performed each assessment, blinding status, and patient independence in questionnaire completion.

### Statistical analysis

All statistical analyses were performed using SPSS version 26.0 (IBM Corp., Armonk, NY, USA) and R software (version 4.2.1, packages: emmeans, effectsize). Categorical data were expressed as frequency and percentage, and the χ^2^ test or Fisher exact test was used for group comparisons. Measurement data that conformed to a normal distribution were expressed as mean ± standard deviation (SD). For between-group comparisons of continuous outcomes at baseline, independent-samples *t*-tests were used. To evaluate the intervention effect over time, a 2 (group: study group vs. control group) × 2 (time: baseline vs. 3 months) repeated-measures analysis of variance (RM-ANOVA) was performed for each continuous outcome. The primary focus of the analysis was the Group × Time interaction effect, which indicates whether the change over time differed significantly between the two groups. When a significant interaction was detected, post-hoc simple effects analyses were conducted with Bonferroni correction for multiple comparisons, comparing: (1) within-group changes from baseline to 3 months for each group, and (2) between-group differences at each time point. For each analysis, the following statistics were reported: Exact *p* values, Effect sizes: Cohen’s d for *t*-tests (with 95% confidence intervals); partial eta squared (ηp^2^) for ANOVA interaction effects (interpreted as small: 0.01, medium: 0.06, large: 0.14), and 95% confidence intervals for mean differences between groups.

Given the multiple outcomes and scale dimensions analyzed, we applied the Benjamini-Hochberg false discovery rate (FDR) procedure to control for type I error. The FDR-adjusted significance threshold was set at *q* < 0.05. Results that remained significant after FDR correction are indicated in the tables and figures.

All primary analyses were conducted according to the intention-to-treat (ITT) principle, including all 120 randomized patients in their originally assigned groups, regardless of whether they received the full intervention or completed all follow-up assessments. For missing outcome data at the 3-month follow-up (study group: *n* = 4, 6.7%; control group: *n* = 2, 3.3%), multiple imputation was performed using chained equations with 20 imputed datasets. The imputation model included baseline outcome values, age, sex, and group assignment. Supplementary Table S3 provides a detailed summary of of missing data and handling methods. Statistical significance was set at *p* < 0.05 (two-tailed) before FDR correction. All reported *p* values are two-tailed.

## Results

### Participant flow and follow-up

**Figure 1:**
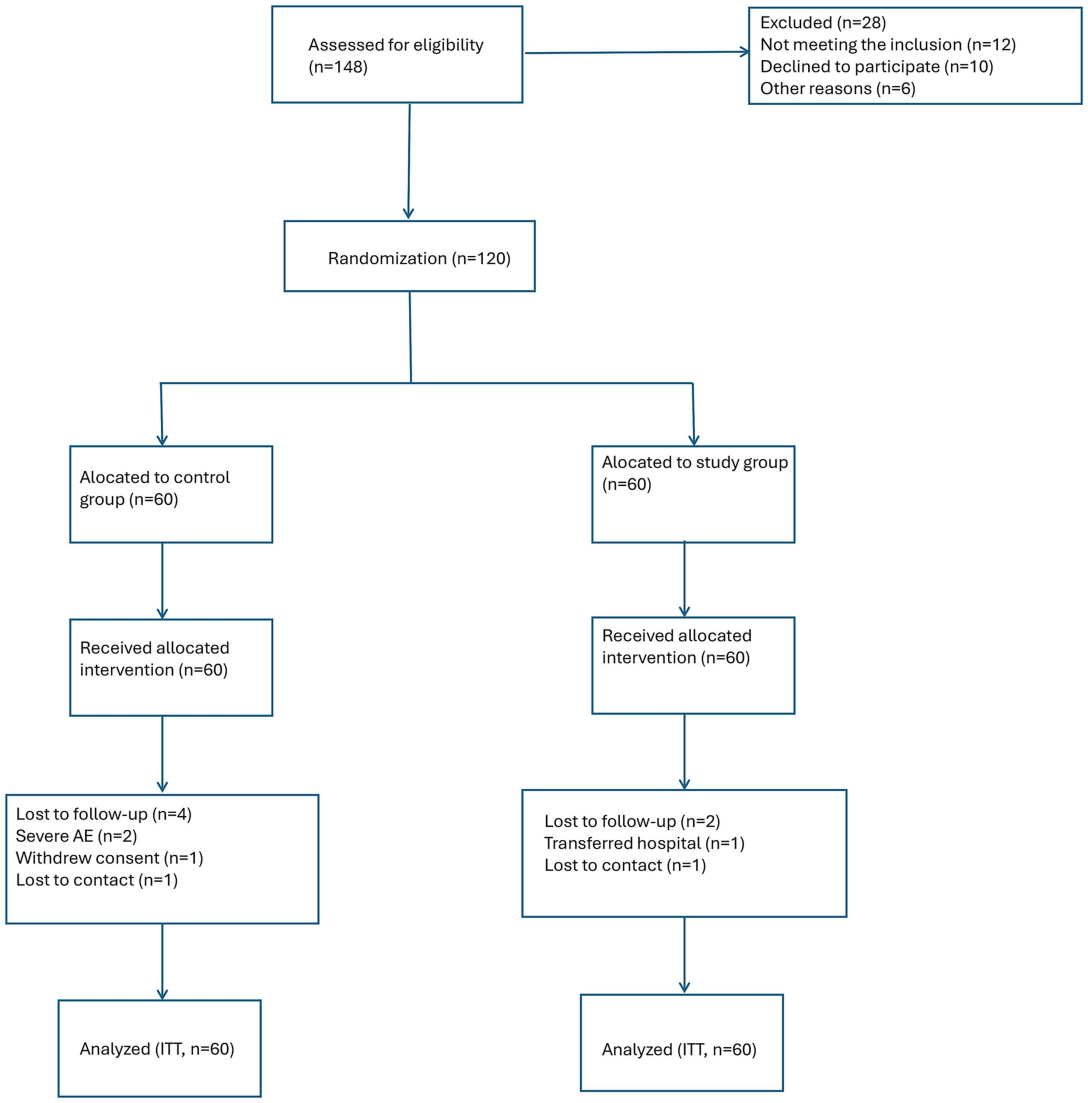
CONSORT flow diagram.

Figure 1 (CONSORT flow diagram) presents the participant flow throughout the trial. Of the 148 patients initially assessed for eligibility, 28 were excluded (12 did not meet inclusion criteria, 10 declined to participate, and 6 were excluded for other reasons). A total of 120 patients were randomized, with 60 allocated to the study group and 60 to the control group.

In the study group, all 60 patients received the allocated intervention as planned. During the 3-month follow-up period, 4 patients (6.7%) discontinued the intervention or were lost to follow-up (2 due to severe chemotherapy-related adverse events requiring hospitalization, 1 due to personal decision to withdraw, and 1 lost to contact). In the control group, 2 patients (3.3%) were lost to follow-up (1 due to transfer to another hospital and 1 lost to contact). Thus, 56 patients in the study group and 58 patients in the control group completed the full 3-month follow-up and were included in the per-protocol analysis. All 120 patients were included in the ITT analysis, with missing outcome data handled using multiple imputation as described above.

No serious adverse events related to the intervention were reported in either group.

### Baseline characteristics of participants

The CONSORT flow diagram (Figure 1) summarizes patient enrollment, randomization, follow-up, and analysis. As shown in Table 1, no significant differences were observed between the two groups in any of the baseline demographic, disease-related, treatment-related, comorbidity, or nutritional variables (all *p* > 0.05), indicating successful randomization and adequate group comparability.

**Table 1:** Baseline demographic and clinical characteristics of the study population.

Variable	Control group (*n* = 60)	Study group (*n* = 60)	*p*
Gender (*n*, %)			0.714
Male	32 (53.33)	30 (50.00)	
Female	28 (46.67)	30 (50.00)	
Age (years, mean ± SD)	48.25 ± 5.62	48.32 ± 5.78	0.946
BMI (kg/m^2^, mean ± SD)	21.54 ± 1.56	21.42 ± 1.46	0.664
TNM stage (*n*, %)			0.927
Stage I	20 (33.33)	22 (36.67)	
Stage II	25 (41.67)	24 (40.00)	
Stage III	15 (25.00)	14 (23.33)	
Educational level (*n*, %)			0.838
Junior high school and below	19 (31.67)	20 (33.33)	
High school/vocational school	20 (33.33)	22 (36.67)	
College degree or above	21 (35.00)	18 (30.00)	
Chemotherapy regimen (*n*, %)			0.988
FOLFOX	30 (50.00)	28 (46.67)	
FOLFIRI	16 (26.67)	18 (30.00)	
XELOX	12 (20.00)	12 (20.00)	
Other	2 (3.33)	2 (3.33)	
Postoperative interval (days, mean ± SD)	29.23 ± 7.92	28.54 ± 8.27	0.641
Ostomy status (present, *n*, %)	16 (26.67)	16 (26.67)	1.000
Number of planned cycles (mean ± SD)	8.45 ± 2.05	8.29 ± 2.12	0.675
Number of completed cycles at baseline (mean ± SD)	1.13 ± 0.42	1.21 ± 0.53	0.361
Vascular access device (*n*, %)			0.976
PICC	37 (61.67)	35 (58.33)	
PORT	14 (23.33)	15 (25.00)	
Peripheral vein	9 (15.00)	10 (16.67)	
Hypertension (*n*, %)	20 (33.3%)	18 (30.0%)	
Diabetes (*n*, %)	10 (16.7%)	12 (20.0%)	0.814
Cardiovascular disease (*n*, %)	9 (15.0%)	8 (13.3%)	>0.999
Charlson Comorbidity Index (mean ± SD)	1.93 ± 1.32	1.87 ± 1.26	0.799
Serum albumin (g/L, mean ± SD)	37.92 ± 4.83	38.27 ± 4.56	0.683
Weight loss in past 3 months (%, mean ± SD)	4.55 ± 3.02	4.28 ± 2.83	0.614

SD, standard deviation; BMI, body mass index; PICC, peripherally inserted central catheter; PORT, implantable venous access port; FOLFOX, fluorouracil + leucovorin + oxaliplatin; FOLFIRI, fluorouracil + leucovorin + irinotecan; XELOX, capecitabine + oxaliplatin.

### Gastrointestinal function

Before intervention, no significant differences were observed in GSRS scores between the two groups (abdominal symptoms: mean difference = 0.35, 95% CI: −0.82 to 1.52, *t*(118) = 0.59, *p* = 0.557, Cohen’s d = 0.11 [95% CI: −0.25 to 0.47]; defecation status: mean difference = 0.22, 95% CI: −0.68 to 1.12, *t*(118) = 0.48, *p* = 0.632, Cohen’s d = 0.09 [95% CI: −0.27 to 0.45]).

**Figure 2:**
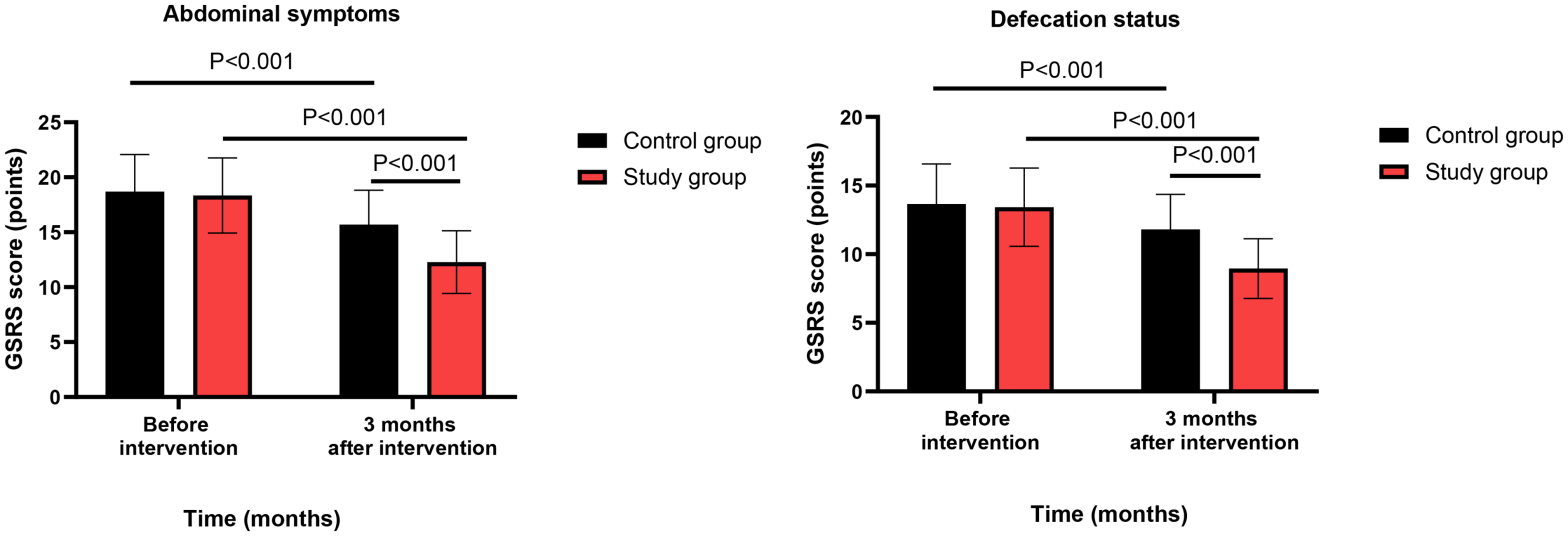
Gastrointestinal function between the two groups.

Three months after intervention, repeated-measures ANOVA revealed significant group-by-time interaction effects for both abdominal symptoms (*F*(1,118) = 24.36, *p* < 0.001, ηp^2^ = 0.171 [95% CI: 0.082 to 0.268], a large effect) and defecation status (*F*(1,118) = 18.92, *p* < 0.001, ηp^2^ = 0.138 [95% CI: 0.056 to 0.228], a medium-to-large effect). Post-hoc simple effects analysis showed that both groups had significant reductions from baseline to 3 months (both *p* < 0.001), but the study group showed a significantly greater reduction than the control group: for abdominal symptoms, mean difference at 3 months = −3.42 (95% CI: −4.85 to −1.99), *t*(118) = −4.71, *p* < 0.001, Cohen’s d = −0.86 [95% CI: −1.23 to −0.49]; for defecation status, mean difference at 3 months = −2.85 (95% CI: −4.12 to −1.58), *t*(118) = −4.41, *p* < 0.001, Cohen’s d = −0.80 [95% CI: −1.17 to −0.43] (Figure 2). All these comparisons remained significant after FDR correction (*q* < 0.01).

### Cancer-related fatigue

Before intervention, no significant differences were observed in CFS scores between the two groups (all *p* > 0.05, Cohen’s d range: 0.05–0.12).

**Figure 3:**
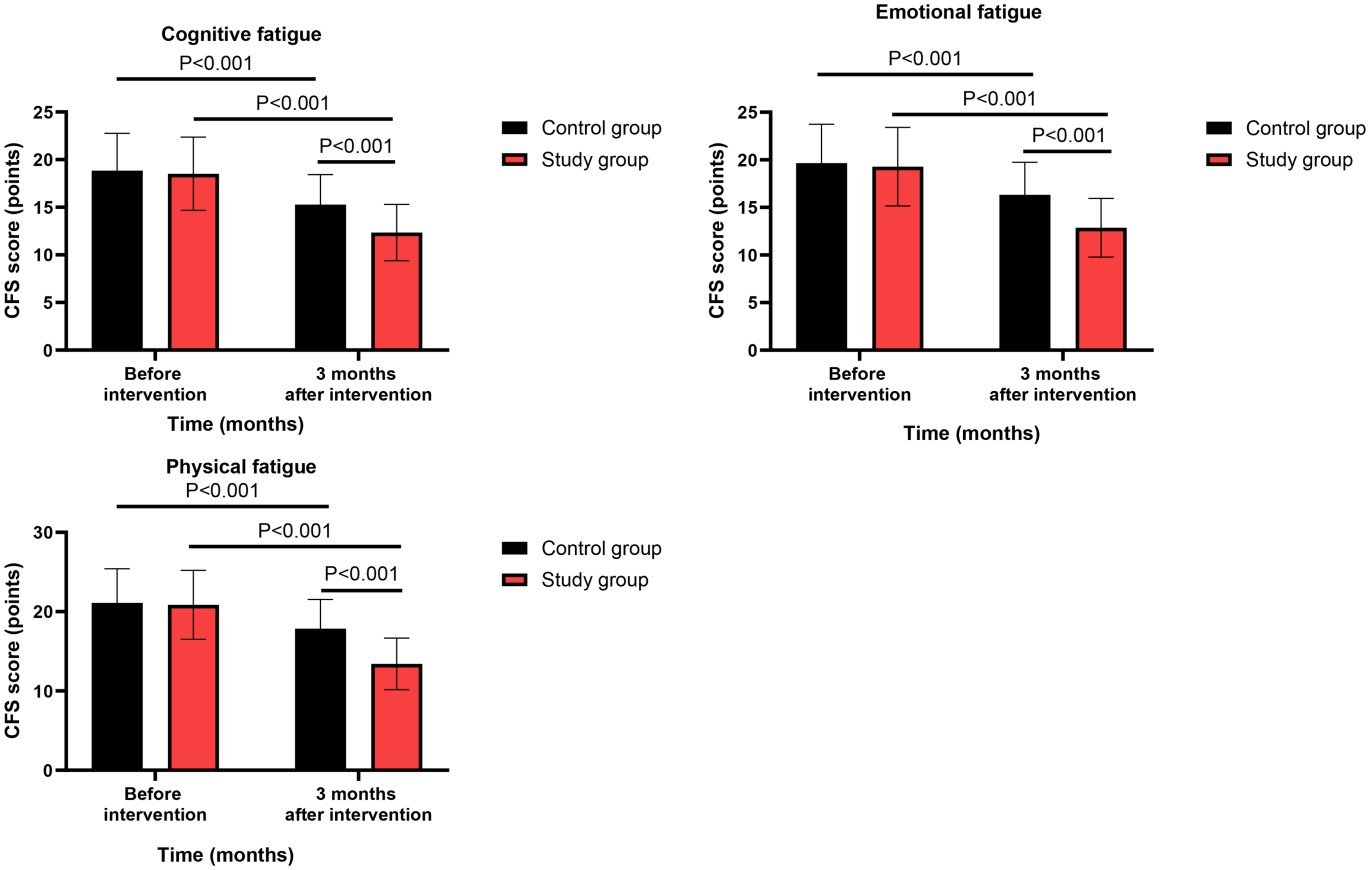
Cancer-related fatigue between the two groups.

Three months after intervention, repeated-measures ANOVA revealed significant group-by-time interaction effects for all three CFS dimensions: cognitive fatigue (*F*(1,118) = 15.23, *p* < 0.001, ηp^2^ = 0.114 [95% CI: 0.041 to 0.199]), emotional fatigue (*F*(1,118) = 20.67, *p* < 0.001, ηp^2^ = 0.149 [95% CI: 0.065 to 0.241]), and physical fatigue (*F*(1,118) = 28.45, *p* < 0.001, ηp^2^ = 0.194 [95% CI: 0.100 to 0.293]). Post-hoc analyses confirmed that the study group had significantly greater reductions than the control group in all three dimensions (all *p* < 0.001, Cohen’s d range: −0.75 to −1.02) (Figure 3). All effects remained significant after FDR correction (*q* < 0.01).

### Psychological resilience

Before intervention, no significant differences were observed in CD-RISC scores between the two groups (all *p* > 0.05, Cohen’s d range: 0.08–0.15).

**Figure 4:**
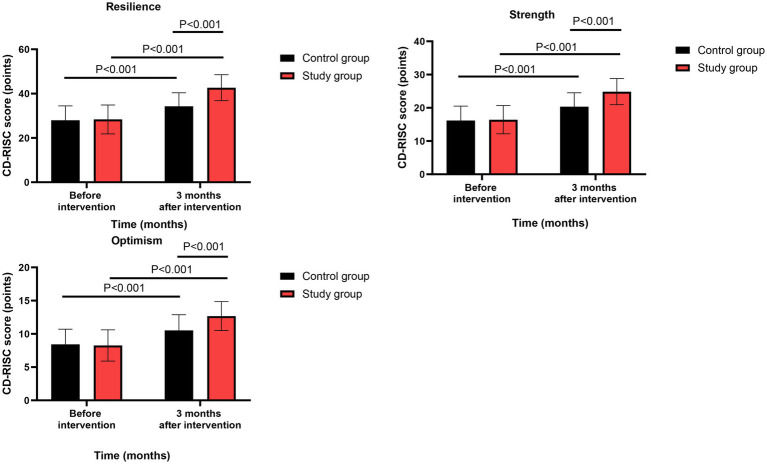
Psychological resilience between the two groups.

Three months after intervention, repeated-measures ANOVA revealed significant group-by-time interaction effects for resilience (*F*(1,118) = 31.82, *p* < 0.001, ηp^2^ = 0.212 [95% CI: 0.113 to 0.314]), strength (*F*(1,118) = 22.46, *p* < 0.001, ηp^2^ = 0.160 [95% CI: 0.073 to 0.253]), and optimism (*F*(1,118) = 14.98, *p* < 0.001, ηp^2^ = 0.113 [95% CI: 0.040 to 0.197]). Post-hoc analyses showed that the study group had significantly greater increases than the control group in all three dimensions (all *p* < 0.001, Cohen’s d range: 0.68–1.10) (Figure 4). All effects remained significant after FDR correction (*q* < 0.01).

### Self-management efficacy

Before intervention, no significant differences were observed in SUPPH scores between the two groups (all *p* > 0.05, Cohen’s d range: 0.07–0.14).

**Figure 5:**
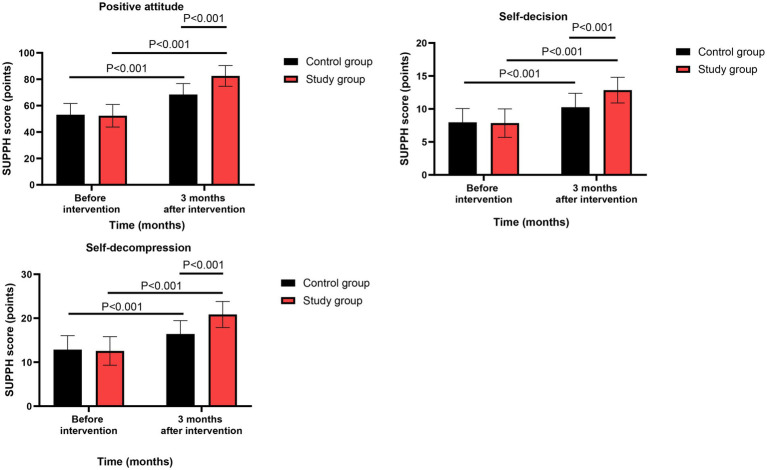
Self-management efficacy between the two groups.

Three months after intervention, repeated-measures ANOVA revealed significant group-by-time interaction effects for positive attitude (*F*(1,118) = 35.67, *p* < 0.001, ηp^2^ = 0.232 [95% CI: 0.130 to 0.337]), self-decision (*F*(1,118) = 19.34, *p* < 0.001, ηp^2^ = 0.141 [95% CI: 0.058 to 0.232]), and self-decompression (*F*(1,118) = 25.78, *p* < 0.001, ηp^2^ = 0.179 [95% CI: 0.087 to 0.278]). Post-hoc analyses confirmed that the study group had significantly greater improvements than the control group in all three dimensions (all *p* < 0.001, Cohen’s d range: 0.72–1.15) (Figure 5). All effects remained significant after FDR correction (*q* < 0.01).

### Quality of life

Before intervention, no significant differences were observed in WHOQOL-BREF scores between the two groups (all *p* > 0.05, Cohen’s d range: 0.06–0.13).

**Figure 6:**
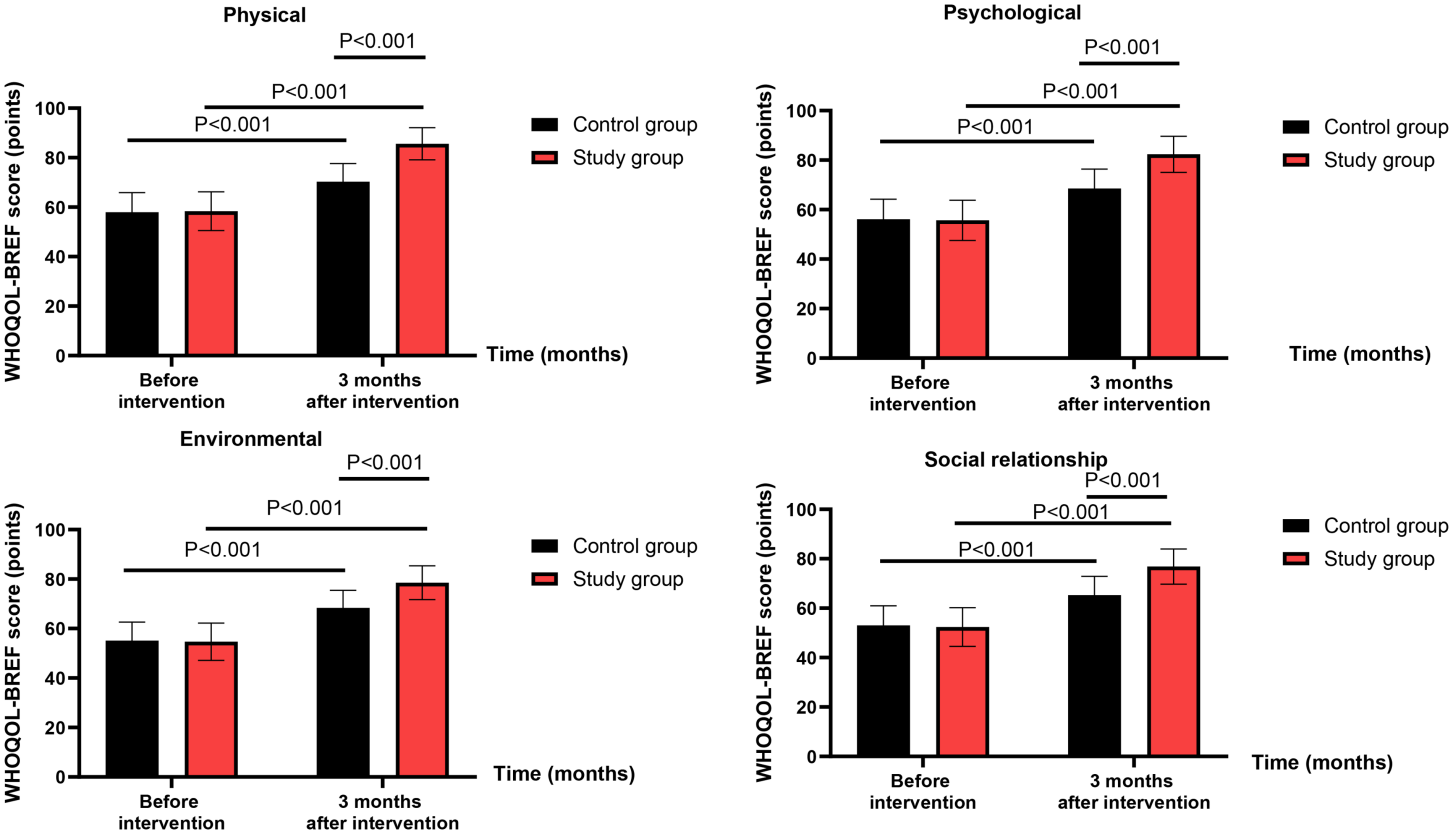
Quality of life between the two groups.

Three months after intervention, repeated-measures ANOVA revealed significant group-by-time interaction effects for physical domain (*F*(1,118) = 42.15, *p* < 0.001, ηp^2^ = 0.263 [95% CI: 0.157 to 0.371]), psychological domain (*F*(1,118) = 33.28, *p* < 0.001, ηp^2^ = 0.220 [95% CI: 0.120 to 0.322]), social relationship domain (*F*(1,118) = 20.93, *p* < 0.001, ηp^2^ = 0.151 [95% CI: 0.066 to 0.243]), and environmental domain (*F*(1,118) = 18.65, *p* < 0.001, ηp^2^ = 0.136 [95% CI: 0.054 to 0.226]). Post-hoc analyses showed that the study group had significantly greater improvements than the control group in all four domains (all *p* < 0.001, Cohen’s d range: 0.69–1.25) (Figure 6). All effects remained significant after FDR correction (*q* < 0.01).

### Incidence of chemotherapy-related adverse reactions

Compared with the control group, the incidence of chemotherapy-related adverse reactions in the study group was lower (8.33% vs. 23.33%; risk difference = −15.00% [95% CI: −27.45% to −2.55%]; χ^2^ = 5.065, *p* = 0.024; odds ratio = 0.30 [95% CI: 0.10 to 0.87]; number needed to treat (NNT) = 6.67 [95% CI: 3.64 to 39.22]) (Table 2). This result remained significant after FDR correction (*q* = 0.036).

**Table 2:** Incidence of chemotherapy-related adverse reactions between the two groups.

Groups	Case	Leukopenia	Gastrointestinal reactions	Liver dysfunction	Total incidence rate
Control group	60	5 (8.33)	6 (10.00)	3 (5.00)	14 (23.33)
Study group	60	2 (3.33)	1 (1.67)	2 (3.33)	5 (8.33)
χ^2^					5.065
*p*					0.024

### Medication adherence

Compared with the control group, medication adherence in the study group was better (95.00% vs. 80.00%; risk difference = 15.00% [95% CI: 4.18 to 25.82%]; χ^2^ = 6.171, *p* = 0.013; odds ratio = 4.75 [95% CI: 1.27 to 17.75]; number needed to treat (NNT) = 6.67 [95% CI: 3.87 to 23.92]) (Table 3). This result remained significant after FDR correction (*q* = 0.026).

**Table 3:** Medication adherence between the two groups.

Groups	Case	Complete adherence	Basic adherence	Non-adherence	Medication adherence rate
Control group	60	23 (38.33)	25 (41.67)	12 (20.00)	48 (80.00)
Study group	60	27 (45.00)	30 (50.00)	3 (5.00)	57 (95.00)
χ^2^					6.171
*p*					0.013

## Discussion

This study demonstrates that a structured, multidisciplinary collaborative continuous care model significantly improves a wide range of patient-reported and clinical outcomes in CRC patients undergoing chemotherapy, compared to routine care, over a 3-month follow-up period. Patients in the intervention group exhibited superior gastrointestinal function, reduced cancer-related fatigue, enhanced psychological resilience and self-management efficacy, better quality of life, fewer chemotherapy-related adverse reactions, and higher medication adherence after the 3-month intervention period. These findings should be interpreted as evidence of short-term effectiveness of this supportive care model.

The multifaceted benefits observed can be attributed to the holistic and proactive nature of the intervention, which directly addressed the limitations of conventional, symptom-focused follow-up. Firstly, the significant reduction in GSRS scores suggests improved management of chemotherapy-induced gastrointestinal toxicity. This was likely achieved through the combined effects of individualized dietary guidance from the nutritionist, proactive symptom monitoring via the electronic system enabling early intervention, and detailed, personalized education on medication side effects. This comprehensive therapy is no longer limited to general advice. It can regulate the intestinal microbiota, thereby better controlling the symptoms.

Secondly, the marked decrease in cancer-related fatigue (CFS scores) is a critical finding. Fatigue in this population is multifactorial, stemming from the disease itself, chemotherapy, anemia, psychological distress, and poor sleep (14). Our model attacked these contributors simultaneously: physical activity guidance from the rehabilitation team countered deconditioning; psychological counseling addressed emotional exhaustion and maladaptive cognitions; nutritional optimization helped correct anemia and maintain energy levels; and effective symptom management reduced the overall burden of discomfort. This multi-pronged strategy aligns with current understanding that fatigue management requires a comprehensive, not singular, approach (15).

Thirdly, the enhancement in psychological resilience (CD-RISC) and self-management efficacy (SUPPH) highlights the model’s success in empowering patients. Continuous access to a specialized resource team (psychologist, specialist nurses) provided patients with tools for cognitive reframing, stress reduction (e.g., yoga, sandplay), and problem-solving skills. The peer support fostered in the rehabilitation club reduced feelings of isolation and provided vicarious learning experiences. By transforming patients from passive recipients of care into informed, active managers of their health, the intervention likely increased their sense of control and self-efficacy, which are key determinants of psychological adaptation and adherence (16).

Consequently, the superior quality of life (WHOQOL-BREF) scores across physical, psychological, social, and environmental domains represent the synergistic culmination of these improvements. Better-controlled symptoms, less fatigue, stronger mental fortitude, and an active support network directly translate into enhanced daily functioning and well-being (17). The lower incidence of adverse reactions and higher medication adherence further underscore the model’s clinical impact. Adherence likely improved due to better understanding of drug purposes (from tailored education), reduced fear of side effects (through proactive management), and the ongoing support and accountability provided by the follow-up and volunteer teams.

Our findings strongly corroborate and extend previous research on multidisciplinary care and continuous care in oncology. Prior studies have shown that nurse-led or multidisciplinary interventions can improve quality of life and reduce symptom burden in cancer patients (18). Similarly, Huang et al. suggested that multidisciplinary team collaborative nursing enhances treatment adherence and quality of life, and reduces the incidence of complications in patients undergoing gastrointestinal tumor surgery (19). In addition, Zhang et al. indicated that continuous care is associated with reduced cancer-related fatigue, improved self-care ability, low fear of recurrence and few complications in patients receiving intravesical chemotherapy after transurethral resection of bladder tumor (20).

However, our study makes several distinct contributions. First, while many existing models are implemented in outpatient clinic settings, our intervention seamlessly bridged the hospital-to-home transition, a critically vulnerable period. The deployment of community nurses and volunteers for home visits and the use of digital platforms (WeChat, QQ) ensured continuity and accessibility, addressing a common gap in post-discharge care. Second, we provided a more granular evaluation by measuring psychological resilience and self-management efficacy as specific mediators of outcome improvement, rather than merely reporting global quality of life. This offers deeper insight into how the intervention worked. Third, the inclusion of medication adherence as a key outcome links the supportive care model directly to a crucial clinical behavior that influences treatment efficacy and survival.

Most notably, our results on significantly reducing chemotherapy-related adverse reactions add a valuable clinical dimension. While supportive care aims to improve well-being, demonstrating a tangible reduction in complications like leukopenia or liver dysfunction suggests that such comprehensive management may also have a modulatory effect on treatment tolerance and potentially on host defense mechanisms. This aligns with emerging research on the biobehavioral interface, where psychological stress and poor self-care may exacerbate treatment toxicity (21, 22).

### Limitations and future directions

This study has several limitations that should be considered when interpreting the findings.

First, the lack of blinding for participants and intervention providers represents a significant source of potential bias. Due to the nature of the intervention—multidisciplinary collaborative continuous care involving active patient engagement, education sessions, home visits, and social media group participation—it was not feasible to blind participants or the nursing staff delivering the intervention to group assignment. This introduces two related but distinct sources of bias:

*Performance bias*: Participants who knew they were receiving the active intervention may have changed their behavior or reported outcomes differently (e.g., reporting better quality of life because they expected to benefit). Conversely, participants in the control group who received only routine care may have experienced disappointment or demoralization, potentially exaggerating between-group differences.

*Detection bias*: Although outcome assessors were blinded to group assignment and patients completed all questionnaires independently in a private room using sealed opaque envelopes, patients themselves were aware of their group allocation. For patient-reported outcomes (GSRS, CFS, CD-RISC, SUPPH, WHOQOL-BREF, and medication adherence), this awareness may have influenced responses through expectation effects or social desirability bias.

The potential direction of these biases is likely toward overestimation of the intervention’s effectiveness. Participants receiving the active intervention may have reported more favorable outcomes due to positive expectations, while control participants may have reported less favorable outcomes due to disappointment. Therefore, the observed effect sizes should be interpreted cautiously as they may represent an upper bound of the true intervention effect.

Several strategies were implemented to mitigate these biases as much as possible: (1) outcome assessors were blinded to group allocation; (2) patients completed all questionnaires independently without the presence of any nursing staff or assessor; (3) completed questionnaires were placed into sealed opaque envelopes before collection; (4) objective outcomes (chemotherapy-related adverse reactions) were extracted from electronic medical records by a blinded research assistant, and these results were consistent with the patient-reported outcomes; (5) the statistician was blinded until final analysis completion. The consistency between subjective patient-reported outcomes and objective clinical data (adverse reactions) provides some reassurance that the findings are not solely attributable to bias.

Second, the most significant limitation is the short follow-up duration of 3 months. While this period is sufficient to evaluate short-term symptom burden, psychological improvement, and early adherence, it is not adequate to support conclusions regarding long-term quality of life, sustained adherence, treatment completion rates, rehospitalization, disease recurrence, or overall survival. The observed improvements may diminish over time without continued support. Therefore, our findings should be interpreted as evidence of short-term effectiveness.

Third, the study was conducted at a single center, which may limit generalizability to other healthcare settings with different resources, patient populations, and healthcare systems.

Fourth, the dropout rate in the study group (6.7%) was slightly higher than in the control group (3.3%), which may introduce some attrition bias. However, sensitivity analyses comparing ITT and complete-case analyses showed consistent results, suggesting that the impact of attrition was minimal.

Fifth, the study analyzed multiple secondary outcomes without adjusting the sample size for multiple comparisons. Although we applied FDR correction to control type I error, the sample size was calculated based solely on the primary outcome, meaning that the study may be underpowered to detect small effects for secondary outcomes.

Future research should: (1) consider using sham interventions or attention-matched control groups to reduce expectation effects and better isolate the specific effects of the intervention; (2) extend the follow-up period to at least 12 months or longer to evaluate durability of intervention effects; (3) conduct multi-center randomized trials to enhance generalizability; (4) explore the physiological mechanisms underlying the observed improvements; and (5) conduct cost–benefit analyses to facilitate wider promotion. Until sham-controlled or attention-matched trials are conducted, the possibility that some of the observed benefits are attributable to expectation effects rather than the specific intervention components cannot be excluded.

## Conclusion

In conclusion, this multidisciplinary collaborative continuous care model, integrating clinical, nutritional, psychological, and social support through a structured team and digital tools, proved effective in optimizing the comprehensive recovery of CRC patients during chemotherapy over a 3-month period. It ameliorated physical and psychological symptoms, empowered patients, improved short-term treatment adherence and tolerance, and enhanced quality of life in the short term. This study provides evidence for moving beyond reactive, symptom-based follow-up toward a proactive, holistic, and integrated standard of supportive care in oncology during the early post-discharge phase. However, given the short follow-up duration, these findings should be considered preliminary evidence of short-term effectiveness. Longer-term studies are necessary to determine whether these benefits are sustained and translate into improved clinical outcomes such as treatment completion, reduced rehospitalization, and lower recurrence rates.

## Data Availability

The original contributions presented in the study are included in the article/Supplementary material, further inquiries can be directed to the corresponding author.
