# Supplementary material for: Impact of continuous care based on multidisciplinary collaboration on the quality of life of patients with colorectal cancer undergoing chemotherapy
Source: Front Med (Lausanne). 2026 Apr 24;13:1799635. doi: 10.3389/fmed.2026.1799635 (PMC13154399; doi:10.3389/fmed.2026.1799635)
Supplement: Supplementary file 1 [file Table_1.docx]

Table S1 Summary of intervention components: planned frequency, actual delivery, completion rates, and patient engagement metrics

| Intervention component | Planned frequency / dose | Actual delivery | Completion rate (%) | Patient engagement / adherence | Quality control notes |
| --- | --- | --- | --- | --- | --- |
| **Clinical team** |  |  |  |  |  |
| Daily inpatient nursing | Daily during hospitalization (mean LOS: 6.3±1.8 days) | Delivered to all 60 patients | 100% | N/A (routine care) | Documented in nursing records |
| Discharge education session | One 30–40 min session on discharge day | Delivered to all 60 patients | 100% | All patients received printed + electronic handbook | Checklist completed for each patient |
| Ostomy hands-on training | One 30-min session with return demonstration | Delivered to 32/32 patients with ostomy | 100% (among ostomy patients) | 100% demonstrated correct bag change | Skills checklist signed off |
| **Follow-up team** |  |  |  |  |  |
| Telephone follow-up (24 h) | Once within 24 h post-discharge | 60/60 completed | 100% | N/A | Call log recorded |
| Telephone follow-up (week 2) | Once at week 2 | 60/60 completed | 100% | N/A | Call log recorded |
| Telephone follow-up (week 4) | Once at week 4 | 60/60 completed | 100% | N/A | Call log recorded |
| Telephone follow-up (week 8) | Once at week 8 | 59/60 completed | 98.3% | One patient unreachable after 3 attempts | Call log recorded |
| Telephone follow-up (week 12) | Once at week 12 | 58/60 completed | 96.7% | Two patients unreachable | Call log recorded |
| WeChat/QQ group health posts | Weekly (12 posts over 3 months) | 12/12 posts delivered | 100% | Average active readership*: 78.3% (range 65–88%) | Screenshots retained; monthly summary |
| Patient enrollment in groups | Required | 60/60 enrolled | 100% | Average patient-initiated messages/week: 3.2 (range 0–12) | Enrollment log |
| **Resource team** |  |  |  |  |  |
| Weekly educational course | Weekly (12 courses over 3 months; 45–60 min each) | 12/12 courses delivered | 100% | In-person attendance: average 51.2/60 (85.3%) | Attendance sheet; video recording |
| Video push to groups | Within 3 days post-course | 12/12 videos pushed | 100% | Average view rate within 1 week: 71.2% (range 62–81%) | Platform analytics |
| Specialist consultation (as needed) | On demand | 18 consultations for 14 patients | N/A | 14/60 patients (23.3%) received ≥1 consultation | Consultation log |
| **Volunteer team** |  |  |  |  |  |
| Home visits | Once every 2 weeks (6 visits/patient over 3 months) | 342/360 visits completed | 95.0% | Average visit duration: 36.4±7.2 min | Sign-in sheet; visit log |
| Missed visits |  | Patient refusal: 12 visits (3.3%) |  |  |  |
| Missed visits |  | Scheduling conflict: 6 visits (1.7%) |  |  |  |
| **Rehabilitation club team** |  |  |  |  |  |
| Psychological counseling sessions (sandplay, yoga, face-to-face) | Weekly (12 sessions over 3 months) | 12/12 sessions held | 100% | Average attendance: 42.3/60 (70.5%) | Attendance sheet |
| Fellowship meetings | Monthly (3 meetings over 3 months) | 3/3 meetings held | 100% | Average attendance: 38.7/60 (64.5%) | Attendance sheet |
| **Overall** |  |  | 94.8%† |  |  |

LOS: length of stay; N/A: not applicable.

Active readership defined as clicking “like” or replying to the post within 48 hours.

† Overall completion rate calculated as (total actual deliveries / total planned deliveries) across all discrete countable components (excluding as-needed consultations).
