# Supplementary material for: Impact of continuous care based on multidisciplinary collaboration on the quality of life of patients with colorectal cancer undergoing chemotherapy
Source: Front Med (Lausanne). 2026 Apr 24;13:1799635. doi: 10.3389/fmed.2026.1799635 (PMC13154399; doi:10.3389/fmed.2026.1799635)
Supplement: Supplementary file 2 [file Table_2.docx]

Table S2. Summary of outcome assessment procedures

| Outcome measure | Assessor | Blinding to group allocation | Patient independence | Quality control |
| --- | --- | --- | --- | --- |
| GSRS (gastrointestinal function) | Trained assessor from Follow-up team (not involved in prior patient communication) | Yes | Self-completed, sealed envelope | Standardized script; ICC=0.94 |
| CFS (cancer-related fatigue) | Same as above | Yes | Self-completed, sealed envelope | Standardized script; ICC=0.94 |
| CD-RISC (psychological resilience) | Same as above | Yes | Self-completed, sealed envelope | Standardized script; ICC=0.94 |
| SUPPH (self-management efficacy) | Same as above | Yes | Self-completed, sealed envelope | Standardized script; ICC=0.94 |
| WHOQOL-BREF (quality of life) | Same as above | Yes | Self-completed, sealed envelope | Standardized script; ICC=0.94 |
| Morisky Medication Adherence | Same as above | Yes | Self-completed, sealed envelope | Standardized script; ICC=0.94 |
| Chemotherapy-related adverse reactions (leukopenia, GI reactions, liver dysfunction) | Research assistant (blinded) extracting from EMR | Yes | N/A (objective data) | Pre-specified criteria; double extraction for 20% of cases |

GSRS: Gastrointestinal Symptom Rating Scale; CFS: Cancer Fatigue Scale; CD-RISC: Connor-Davidson Resilience Scale; SUPPH: Strategies Used by People to Promote Health; WHOQOL-BREF: World Health Organization Quality of Life Scale-Brief; EMR: electronic medical record; GI: gastrointestinal; ICC: intraclass correlation coefficient.
