# Supplementary material for: Impact of continuous care based on multidisciplinary collaboration on the quality of life of patients with colorectal cancer undergoing chemotherapy
Source: Front Med (Lausanne). 2026 Apr 24;13:1799635. doi: 10.3389/fmed.2026.1799635 (PMC13154399; doi:10.3389/fmed.2026.1799635)
Supplement: Supplementary file 3 [file Table_3.docx]

Table S3. Summary of missing data and handling methods

| Outcome measure | Study group (n=60) | Control group (n=60) | Missing data handling |
| --- | --- | --- | --- |
| GSRS | 4 missing (6.7%) | 2 missing (3.3%) | Multiple imputation (20 datasets) |
| CFS | 4 missing (6.7%) | 2 missing (3.3%) | Multiple imputation (20 datasets) |
| CD-RISC | 4 missing (6.7%) | 2 missing (3.3%) | Multiple imputation (20 datasets) |
| SUPPH | 4 missing (6.7%) | 2 missing (3.3%) | Multiple imputation (20 datasets) |
| WHOQOL-BREF | 4 missing (6.7%) | 2 missing (3.3%) | Multiple imputation (20 datasets) |
| Morisky Adherence | 4 missing (6.7%) | 2 missing (3.3%) | Multiple imputation (20 datasets) |
| Adverse reactions | 0 missing (0%) | 0 missing (0%) | Complete data (EMR extraction) |

ITT: intention-to-treat; EMR: electronic medical record; GSRS: Gastrointestinal Symptom Rating Scale; CFS: Cancer Fatigue Scale; CD-RISC: Connor-Davidson Resilience Scale; SUPPH: Strategies Used by People to Promote Health; WHOQOL-BREF: World Health Organization Quality of Life Scale-Brief.
